# Design, Construction, and Evaluation of a Combined Atmospheric Cold Plasma‐Pulsed Electric Field Spraying System for Pasteurization of Sour Cherry Juice

**DOI:** 10.1002/fsn3.70465

**Published:** 2025-06-23

**Authors:** Farhad Jamali‐Hafshejani, Bahram Hosseinzadeh Samani, Kimia Taki, Shirin Ghatrehsamani

**Affiliations:** ^1^ Department of Mechanical Engineering of Biosystems Shahrekord University Shahrekord Iran; ^2^ Department of Agricultural and Biological Engineering The Pennsylvania State University University Park Pennsylvania USA

**Keywords:** atmospheric pressure cold plasma, *Escherichia coli*, nonthermal technology, pulsed electric field, sour cherry juice

## Abstract

This study developed a nonthermal pasteurization method for heat‐sensitive sour cherry juice using hurdle technology, combining atmospheric cold plasma (ACP) and pulsed electric field (PEF) to reduce microbial load while preserving bioactive compounds. The effects of PEF intensity (5–10 kV/cm), exposure time (5–35 s), argon‐to‐air ratio (0–1), and plasma jet‐nozzle angle (0°–90°) on *Escherichia coli* inactivation in the ACP‐PEF system were assessed. Exposure time was the dominant factor in *E. coli* reduction. Optimal conditions of PEF intensity of 10 kV/cm, 35 s exposure, 0.63 argon‐to‐air ratio, and 3.44° jet‐nozzle angle yielded a 5.73‐log *E. coli* reduction. Compared to individual PEF or ACP treatments, the combined system showed a synergistic effect, enhancing microbial inactivation. Compared to conventional thermal processing, ACP‐PEF better preserved juice quality, with minimal changes in total phenolic compounds (TPC), anthocyanin content (TAC), ascorbic acid, and color indices. This integrated approach ensures effective microbial control while maintaining sour cherry juice's sensory and nutritional attributes, offering a promising alternative to traditional methods.

## Graphical abstract

A combined atmospheric cold plasma–pulsed electric field (ACP‐PEF) system was developed to pasteurize sour cherry juice nonthermally.

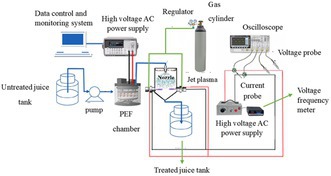

## 1 Introduction

Foodborne illnesses pose a significant burden on public health systems. Pathogens transmitted through food can lead to infectious conditions, causing substantial morbidity and mortality across populations, particularly among infants and young children, women who are pregnant, older individuals, and those with compromised immune function (Luksiene 2021). The majority of foods are vulnerable to contamination at various stages of the supply chain, from production to consumption. Thus, regulating microbial contamination within the food industry is paramount to guaranteeing both product security and excellence (Sakudo 2017). Sour cherry ( *Prunus cerasus* L.) is a globally significant horticultural crop, widely processed into juice and other products due to its rich antioxidant and anti‐inflammatory compounds, including phenolic compounds and vitamins (Toydemir et al. 2013). However, sour cherry juice is susceptible to contamination by pathogens such as *Escherichia coli* , posing risks to food safety and public health, particularly for vulnerable populations.

Conventional thermal pasteurization methods, such as heating and canning, effectively control microbial contamination but often degrade heat‐sensitive nutrients, alter sensory properties, and reduce the nutritional quality of fruit juices (Hosseini et al. 2020). These limitations have driven research toward nonthermal technologies that ensure microbial safety while preserving product quality. These alternative methods include atmospheric cold plasma (ACP), pulsed electric field (PEF), high hydrostatic pressure (HHP), radiation, and ultrasound, and aim to achieve similar levels of microbial safety with improved product quality (Dey et al. 2016).

PEF technology represents a nonthermal approach to preserving food. Employing brief electrical pulses to inactivate microorganisms helps extend shelf life while maintaining most of the food's original quality (Yue et al. 2020). In PEF technology, microbial reduction is accomplished by exposing the product to high‐voltage electric pulses delivered in short bursts. Typically ranging from 100–300 V/cm to 20–80 kV/cm and applied for milliseconds to microseconds, this field disrupts microorganisms without relying on heat (Ghoshal 2023). PEF technology offers an advantage over traditional heat treatments for liquid or semisolid foods. It inactivates microorganisms while more effectively preserving unprocessed food's original color, flavor, texture, and nutritional content (Eissa 2012).

Matter can exist in a fourth state, known as plasma (Misra et al. 2016). Energy must be added to ionize the gas to create plasma from a gas. This energy can be provided through heat, electricity, laser light, radiation, or very fast compression. Plasma then exists as a cloud of energetic particles, holding the supplied energy. When these particles recombine, they release the stored energy as visible light and ultraviolet (UV) radiation during the recombination process (Niemira 2012). ACP generates reactive oxygen and nitrogen species (RONS) utilizing gases like argon and air at ambient pressure. This collection of reactive molecules, which includes free radicals and ions, is highly effective at disrupting the cellular structure of bacteria, particularly *E. coli* , resulting in their deactivation. A key benefit of ACP is its minimal impact on product temperature, unlike heat pasteurization. This gentler approach leads to improved retention of delicate compounds in sour cherry juice, such as anthocyanins and ascorbic acid (Hosseini et al. 2020).

Based on studies, both modern nonthermal technologies, PEF and ACP, can be considered suitable for inactivating *E. coli* in liquid food products (Li et al. 2013; Surowsky et al. 2014). Moreover, within the hurdle approach, combining these two techniques with other nonthermal methods has proven effective in inactivating *E. coli* at lower treatment intensities compared to when each is used alone (Gomez‐Gomez et al. 2021; Mahnot et al. 2019). One of the weaknesses of disinfection with PEF is the resistance of spore bacteria to this treatment (Artés‐Hernández et al. 2021). Prior research has demonstrated the ability of cold plasma to deactivate spore bacteria, suggesting that the outer structure of these microorganisms is a primary target of this process (Ding et al. 2022). It is important to highlight that the impact of plasma is limited to the surface layers (Surowsky et al. 2014); so, the smaller the dimensions of the samples used, the more complete disinfection can be expected compared to their larger dimensions.

This study aimed to develop an integrated system grounded in the hurdle technology concept, considering the strengths and limitations of two advanced nonthermal methods, ACP and PEF, for *E. coli* inactivation in sour cherry juice and to assess their impact on its chemical and qualitative attributes.

## 2 Materials and Methods

### 2.1 Design and Construction of the PEF Pasteurization System

A standard PEF processing system generally consists of four key components: the pulse generator, the PEF treatment chamber, the fluid handling system, and the control and monitoring equipment (Jin and Zhang 2020). The pulse generator in the PEF system consists of a power source, a capacitor bank, and switches to produce the pulses. Both direct current (DC) and alternating current (AC) power sources are capable of charging the capacitor bank (Eissa 2012). An AC power supply was utilized in this research to minimize initial setup costs. The amount of energy stored in the capacitor is determined using Equation (1).

(1) $$Q=0.5C_{o}V^{2}$$

In this context, *Q* represents the stored energy, *C*_o_ denotes the capacitance, and *V* signifies the charge voltage (Eissa 2012).

The capacitance will also be obtained from Equation (2).

(2) $$C=\frac{\tau }{R}=\frac{\mathrm{\tau \sigma A}}{d}$$

In this context, the following parameters are defined: τ (s) represents the pulse duration, *R* (Ω) is the resistance, *σ* (S/m) is the electrical conductivity of the food, *d* (m) is the distance between the electrodes, and *A* (mm^2^) is the electrode surface area (Eissa 2012).

The distance between the two electrodes within the parallel treatment chamber is affected by the intensity of the pulsed electric field, as indicated in Equation (3)

(3) $$E=\frac{U}{d}$$

In this context, *E* represents the strength of the electric field (kV/cm), *U* denotes the voltage applied (kV), and *D* indicates the separation distance (cm) between the parallel plates (Jin and Zhang 2020).

The PEF total treatment time (*T*_*t*_) is calculated by Equation (4).

(4) $$T_{t}=n_{p}\times n\times \tau_{c}$$

Based on Equation (5), the calculation of pulses per chamber (*n*_p_) can be determined, whereas Equation (6) provides the method to evaluate the time spent in a single chamber (*T*_r_); the PEF total treatment time can be obtained from Equation (7).

(5) $$n_{p}=T_{r}\times f$$

(6) $$T_{r}=\frac{V}{F}$$

(7) $$T_{t}=n\times \tau_{c}\times f\times \frac{V}{F}$$

In this context: *T*_r_ signifies the time spent in a single chamber, *F* refers to the mean flow rate (cm^3^/s), *V* represents the volume of the gap in one chamber (cm^3^), *n*_p_ indicates the count of pulses administered per chamber, *T*_t_ denotes the total treatment time, *τ*_c_ corresponds to the active pulse width (*s*), *f* is the pulse frequency (Hz), and n refers to the total number of treatment chambers (Jin and Zhang 2020).

### 2.2 Design and Construction of the Atmospheric Pressure Cold Plasma Jet Spraying Pasteurization System

This research employed an AC power source, with the electrodes linked to the power source through a high‐voltage cable. The AC power source used in this study is a variable voltage‐frequency unit capable of providing a voltage range between 0 and 20 kV and a frequency range from 6 to 20 kHz.

The plasma generation system consists of two coaxial electrodes and a ring‐shaped dielectric. The inner electrode, made of tungsten, is configured as a straight wire aligned at the center and maintained at a fixed distance from the ring electrode. A thin ceramic tube is a dielectric material with a copper ring electrode mounted on its outer surface. The central tungsten electrode has a diameter of 2 mm and a length of 8.5 cm. Positioned coaxially at a defined distance from it, the ring electrode features a thickness of 1 mm, a length of 1 cm, and an internal radius of 6 mm.

**FIGURE 1:**
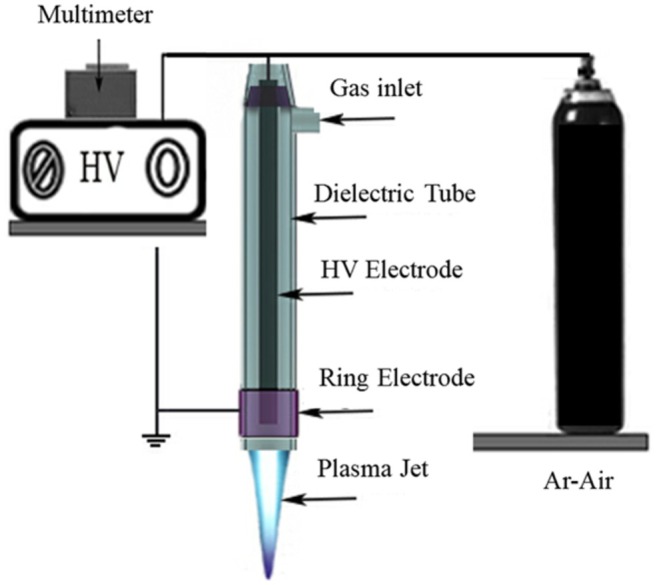
Geometry of the atmospheric pressure cold plasma reactor.

The dielectric thickness is determined by the intensity of the electric field established between the electrodes, which is affected by the voltage and frequency of the power supply (Misra et al. 2016). The geometry of the atmospheric pressure cold plasma reactor is illustrated in Figure 1, showing the coaxial electrode configuration with a single dielectric layer (ceramic tube, thickness 1 mm with an inner radius of 5 mm and a length of 9 cm, placed between the two electrodes) between the tungsten and copper electrodes, ensuring accurate calculation of the electric field intensity.

Equations (8) and (9) will be employed to calculate the electric field intensity across the dielectric layers and the DBD gap. The relationship linking the field strength within these regions is expressed in Equation (10), whereas Equation (11) is used to determine the required voltage for initiating plasma discharge in the DBD system.

(8) $$E_{d}=\frac{V\varepsilon_{g}}{l_{d}\varepsilon_{g}+l_{g}\varepsilon_{d}}$$

(9) $$E_{g}=\frac{V\varepsilon_{d}}{l_{d}\varepsilon_{g}+l_{g}\varepsilon_{d}}$$

(10) $$\frac{E_{d}}{E_{g}}=\frac{\varepsilon_{d}}{\varepsilon_{g}}$$

(11) $$V=l_{d}E_{d}+l_{g}E_{g}$$

Where, *E*_d_ is the field strength in dielectric layers (kV/cm), *E*_g_ is the field strength in the DBD gap (kV/cm), *ɛ*_d_ is the dielectric coefficient, *ɛ*_g_ is the electrical conductivity constant of the gas used to form plasma (equal to 1), *l*_d_ is the dielectric thickness (cm), *l*_g_ is the DBD gap (cm), and *V* is the applied voltage (kV) (Misra et al. 2016).

### 2.3 Design and Construction of a Combined ACP‐PEF Spraying System

The combined pasteurization system of ACP and PEF for inactivating *E. coli* in sour cherry juice consists of two main parts. Considering the results obtained from preliminary experiments and considering the resistance of spore bacteria to PEF and the positive results of the effect of ACP on this type of bacteria, as well as performing electroporation by PEF and creating a more suitable situation for the activities of reactive plasma species (including free radicals) inside the bacterial cell and damaging its DNA, PEF was used first and then ACP.

**FIGURE 2:**
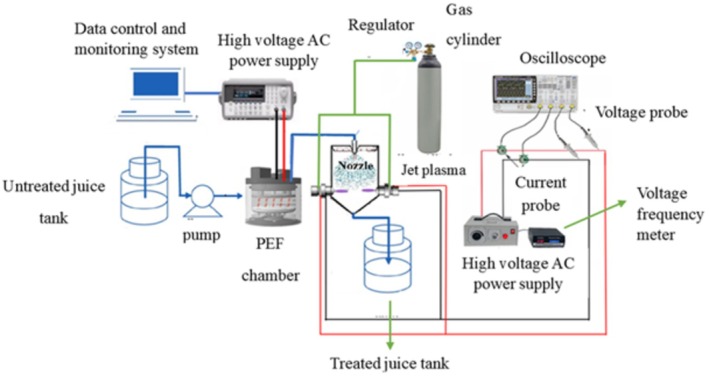
Preliminary design of a combined atmospheric cold plasma‐pulsed electric field spraying system (ACP‐PEF).

After designing and manufacturing the PEF system and the ACP system separately, these two systems were combined according to the design presented in Figure 2, and a diaphragm pump, which is a type of positive displacement pump, was used to transfer cherry juice from the tank to the PEF treatment chamber. After treatment by the PEF system, the sour cherry juice will be sprayed through a nozzle into the ACP treatment chamber, which has two plasma jets with the mentioned specifications. The effect of the plasma jets used was investigated in three different modes. During PEF treatment, a 5–10 kV/cm voltage was applied with a pulse duration of 5–35 s. Due to the nonthermal nature of PEF, any temperature increase from Joule heating was minimal and not directly measured in this study. However, it is expected to be less than 10°C based on similar systems, with potential control via a cooling mechanism in future experiments. An AC power supply with a sinusoidal waveform (frequency 6–20 kHz, voltage 0–20 kV) was used for ACP treatment, consuming approximately 50 W of power. Preliminary assessments suggest RONS concentrations are effective for bacterial inactivation, with electron density and other plasma parameters to be quantified in future spectroscopic analyses.

### 2.4 Preparation of Sour Cherry Juice Samples

Fresh sour cherries were harvested from a local orchard in Chaharmahal and Bakhtiari Province, Iran, for sample preparation. After sorting to eliminate damaged fruits, the cherries were thoroughly washed, cleaned, and pitted. The juice was extracted using a juicer, followed by centrifugation at 4500 rpm for 20 min to separate the pulp from the liquid phase. Subsequently, the clarified juice was filtered using Whatman filter paper under vacuum conditions (Hosseinzadeh Samani et al. 2016).

The processed juice was then transferred into 500 mL glass bottles and divided into two distinct groups. One set was allocated for microbial analysis to evaluate the effectiveness of the ACP‐PEF system in inactivating *E. coli* in sour cherry juice. The second set was reserved for assessing the influence of the ACP‐PEF treatment on the juice's physicochemical and quality parameters.

### 2.5 Microbiological Preparation and Experimental Methodology

To ensure the integrity of microbial testing, all sour cherry juice samples were sterilized prior to inoculation. Sterilization was achieved by autoclaving the samples at 121°C and 15 psi for 15 min to eliminate any preexisting microbial contamination. Following sterilization, the samples were deliberately inoculated with *E. coli* to assess the efficacy of the atmospheric cold plasma‐pulsed electric field (ACP‐PEF) system in reducing bacterial populations. For inoculum preparation, *E. coli* colonies, cultured for 24 h, were transferred into a sterile saline solution (0.9% sodium chloride). A precise volume of this bacterial suspension was then mixed with autoclaved sour cherry juice at a ratio of 1:9 (v/v) to achieve a consistent initial microbial load. Bacterial counts were determined both before and after the ACP‐PEF treatment to evaluate the system's decontamination performance. The viable plate count method was used for microbial enumeration. Each sample underwent a series of five serial dilutions, and 1 mL from each dilution was plated onto MacConkey Agar. The plates were incubated at 37°C for 24 h to allow *E. coli* growth. Post‐incubation, the colonies on each plate were counted to quantify the bacterial concentration in the samples. All experiments were conducted in triplicate to ensure the reproducibility and accuracy of the results (Hosseini et al. 2021).

### 2.6 Determination of TPC and TAC

The TPC and TAC of sour cherry juice were determined following the methods described by (Hosseini et al. 2020) and (Hosseini et al. 2021). Briefly, TPC was quantified using the Folin–Ciocalteu colorimetric assay with gallic acid as the standard, and absorbance was measured at 765 nm. TAC was assessed via the pH differential method, with absorbance recorded at 520 and 700 nm, using cyanidin‐3‐glucoside as the reference. All measurements were performed in triplicate using a UV‐1800 spectrophotometer to ensure precision.

### 2.7 Determination of Vitamin C

The vitamin C content in sour cherry juice was quantified using the iodine titration method as described by (Hosseini et al. 2020). Briefly, juice samples were titrated with a 0.005 mol/L iodine solution in the presence of a starch indicator until the endpoint was reached. Vitamin C concentration was calculated as milligrams per 100 mL of juice. All analyses were conducted in triplicate to ensure accuracy.

(12) $$\text{Ascorbic acid}+I_{2}\to 2\;I^{-}+\text{dehydroascorbic acid}$$

Equation (12) represents I^−^ as iodide ions and I_2_ as iodine (Hosseini et al. 2020).

### 2.8 Measuring Total Color Difference (TDC)

The color attributes of sour cherry juice samples (25 mL, placed in a Petri dish) were evaluated before and after treatment using a colorimeter (Colorflex model, Hunterlab, Virginia, USA). The CIELAB parameters, including L* (lightness), a* (redness), and b* (yellowness), were recorded to quantify color changes. The TDC (Δ*E*) was calculated using the Equation (13).

(13) $$\Delta E=\sqrt{\left({\left(L_{0}-L^{*}\right)}^{2}+{\left(a_{0}-a^{*}\right)}^{2}+{\left(b_{0}-b^{*}\right)}^{2}\right)}$$

In this context, *L*_0_, *a*_0_, and *b*_0_ represent the color measurements of the untreated sour cherry juice samples (Adekunte et al. 2010; Pathare et al. 2013).

### 2.9 Conventional Thermal Method

In order to evaluate sour cherry juice pasteurized using the thermal technique alongside results from the ACP‐PEF system, adjustments were made to simplify the thermal method for practical execution. Specifically, 200 mL of untreated juice was immersed in a hot water bath maintained at 70°C for 1 min (Hosseini et al. 2020). Following this thermal treatment, the sample was cooled, and its TPC, TAC, vitamin C, and TDC were analyzed. The findings were then contrasted with those from sour cherry juice subjected to ACP‐PEF pasteurization under optimal conditions.

### 2.10 Statistical Analysis

The experimental design was structured using Response Surface Methodology (RSM) with a Box–Behnken design to investigate the effects of four independent variables: pulsed electric field (PEF) intensity (5–10 kV/cm), exposure time (5–35 s), argon‐to‐air ratio (0–1), and plasma jet‐nozzle angle (0°–90°). These variables were evaluated at three levels, as outlined in Table 1, resulting in 27 experimental runs with five center point replicates to ensure robustness. The response variables included *E. coli* inactivation, TPC, TAC, vitamin C content, and TCD.

**TABLE 1:** List of independent variables on response surface method.

Independent variable	Range of level		
	−1	0	1
Electric field intensity (kV/cm)	5	7.5	10
Electric field exposure time (s)	5	20	35
Argon/air	0	0.5	1
Angle between plasma jet and nozzle	0	45	90

The general quadratic model for RSM used to analyze the relationships between independent variables and responses is given by Equation (14):

(14) $$Y_{i}=\beta_{0}+\sum \beta_{i}X_{i}+\sum \beta_{\mathrm{ij}}X_{i}X_{j}+\sum \beta_{\mathrm{jj}}{X_{i}}^{2}+\varepsilon$$

In this context, *β*_0_, *β*_*j*_, *β*_*ij*_, and *β*_*jj*_ represent fixed coefficients, whereas *X*_*i*_ and *X*_*j*_ denote independent variables involved in the process, and *ϵ* accounts for unpredictable errors (Hosseini et al. 2021).

Analysis of variance (ANOVA) was applied to determine the significance of main effects and interactions at a 10% probability level (*p* < 0.1). All statistical analyses were conducted using Design Expert to model the responses and optimize the experimental conditions. The design and analysis procedures followed established protocols to ensure reproducibility and accuracy.

## 3 Results and Discussion

### 3.1 Effect of ACP‐PEF Treatment on the Inactivation of *E. coli*

A stepwise regression analysis using analysis of variance (ANOVA) was conducted to assess the impact of process‐related independent variables on the inactivation of *E. coli* . As shown in Table 2, all factors, except for two interactions (1: electric field intensity*angle between the plasma jet and nozzle (AD), 2: electric field exposure time*argon‐to‐air ratio (BC)), as well as the squared term for electric field intensity (A^2^), demonstrated significant effects at a 10% probability level. The lack of fit being insignificant indicates that the model is effective. Based on the ANOVA results, the electric field exposure time emerged as the most influential main variable, explaining roughly 46% of the variation in the data related to *E. coli* inactivation.

**TABLE 2:** The results of ANOVA analysis in response surface method.

Source	df	Sum of squares	Mean square
Model	14	27.76	1.98^a^
A‐ Electric field intensity (kV/cm)	1	10.79	10.79^a^
B‐ Electric field exposure time (s)	1	12.77	12.77^a^
C‐ Argon to air ratio	1	0.1496	0.1496^a^
D‐ Angle between plasma jet and nozzle	1	0.4256	0.4256^a^
AB	1	0.4624	0.4624^a^
AC	1	0.0064	0.0064^a^
AD	1	0.0025	0.0025^ns^
BC	1	0.0000	0.0000^ns^
BD	1	0.0156	0.0156^a^
CD	1	0.0030	0.0030^a^
A^2^	1	0.0001	0.0001^ns^
B^2^	1	0.6211	0.6211^a^
C^2^	1	0.4681	0.4681^a^
D^2^	1	2.57	2.57^a^
Residual	12	0.0096	0.0008
Lack of fit	10	0.0083	0.0008^ns^
Pure error	2	0.0013	0.0006
Cor total	26	27.77	

Abbreviation: ns, not significant.

^a^ Significant effect at 10% level.

The RSM produced a fully quadratic model for predicting the reduction of *E. coli* in sour cherry juice, showing a determination coefficient (*R* ^2^) of 0.9982, a standard deviation (SD) of 0.028, and a coefficient of variation (C.V.) of 0.9. The high *R* ^2^ value reflects a strong correlation between the experimental results and the model's predictions. Similarly, the low C.V. confirms the model's consistency and reliability in simulating the observed data.

Equation (15) represents the relationship derived under coded conditions, whereas Equation (16) depicts the actual model. Among the coefficients in Equation (15), the most significant negative value is associated with the factor of electric field exposure time. Consequently, the constructed model underscores the crucial role of electric field exposure time as the primary variable affecting the effectiveness of the AC‐PEF treatment in inactivating *E. coli* .
(15)

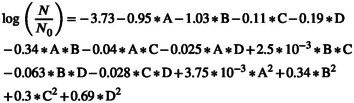



(16)

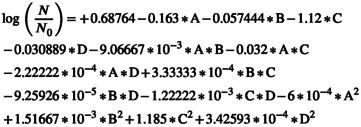




**FIGURE 3:**
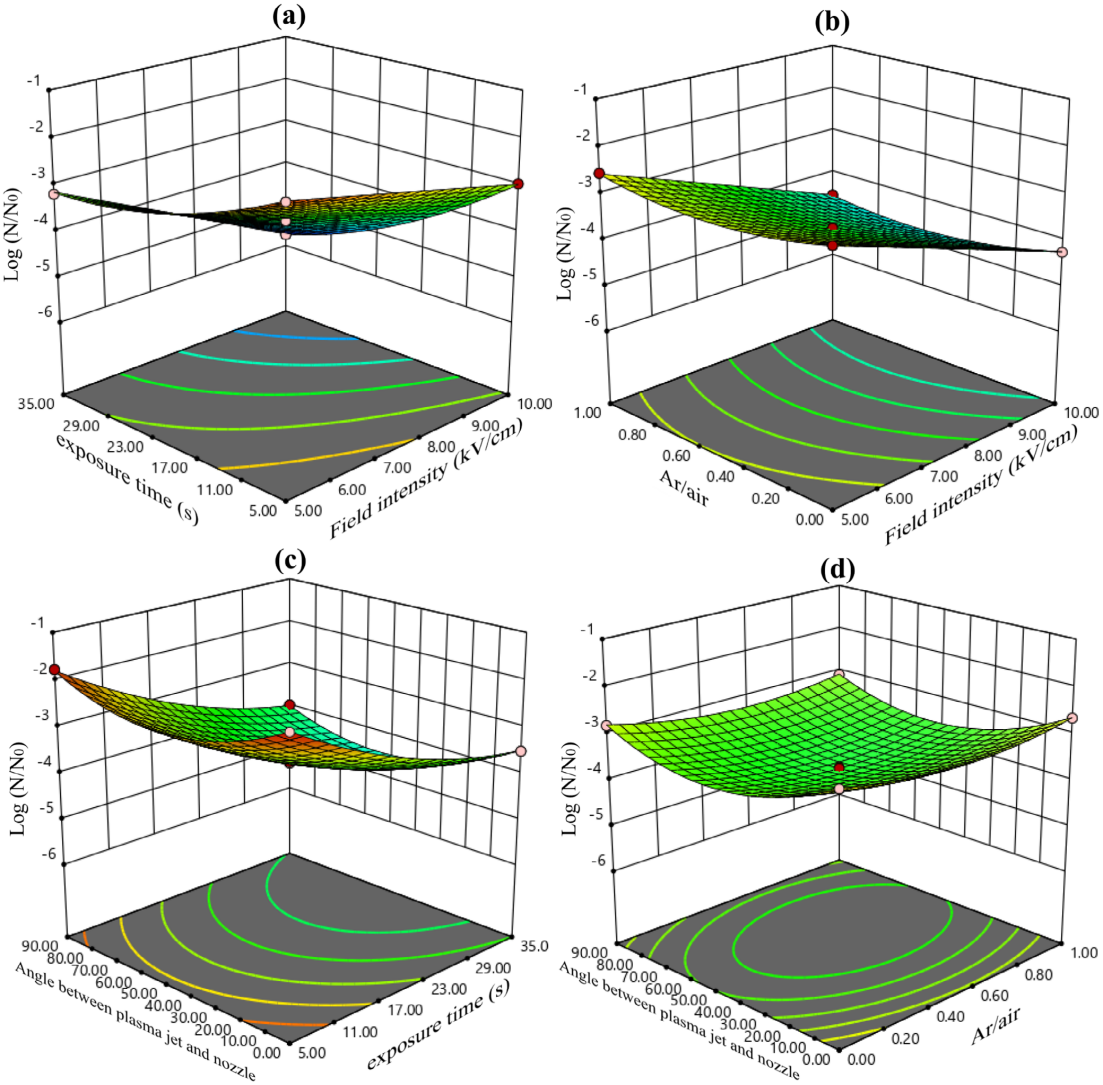
The logarithmic decrease in *E. coli* is affected by (a) electric field intensity and electric field exposure time, (b) electric field intensity and argon to air ratio, (c) electric field exposure time and angle between plasma jet and nozzle, and (d) argon to air ratio and angle between plasma jet and nozzle.

As shown in Figure 3, the reduction of *E. coli* during ACP‐PEF treatment improves with higher electric field intensity, longer electric field exposure time, and greater argon‐to‐air ratios. Among these factors, exposure time has the most pronounced influence. Extending the exposure time from 5 to 35 s led to an 87% increase in bacterial inactivation. In comparison, doubling the electric field intensity from 5 to 10 kV/cm resulted in a 68% increase, whereas adjusting the argon‐to‐air ratio from 0 to 1 only contributed to a 7% enhancement in *E. coli* reduction.

Enhancing the electric field intensity results in a higher rate of membrane disruptions in bacterial cells, thus speeding up their inactivation. A stronger electric field increases both the frequency and speed of electroporation in the membrane, which effectively contributes to reducing microbial levels. Extending the exposure time to the electric field can further enhance *E. coli* inactivation, as prolonged interaction between the field and the liquid allows for more extensive microbial damage. These observations align with previous research that highlights the role of exposure duration in optimizing the effectiveness of nonthermal pasteurization methods (Hosseini et al. 2020; Gomez‐Gomez et al. 2021). However, the findings showed that longer than optimal times can reduce energy efficiency and even negatively impact some product properties.

The ratio of argon to air is crucial for maintaining the stability and quality of the plasma. As an inert gas, argon enhances plasma stability and creates favorable conditions for generating reactive species. However, the results indicated that an excessively high argon‐to‐air ratio can hinder *E. coli* inactivation in sour cherry juice using the ACP‐PEF system. This is because increasing the ratio reduces the concentration of reactive species in the plasma, including ROS and RNS, which are essential for bacterial inactivation (Misra et al. 2016; Yepez et al. 2020; Niemira 2012).

Figure 3c,d shows that by changing the position of the plasma jet and increasing the angle between the plasma jet and the nozzle, the inactivation of *E. coli* by the ACP‐PEF system first increases and then decreases. As the angle between the plasma jets increased from 0° to 45°, the inactivation of bacteria increased by 32%, and then as the angle between the plasma jets increased from 45° to 90°, the inactivation of bacteria decreased by 14%. Thus, by increasing the angle between the plasma jet and the nozzle from 0° to 90°, the inactivation of bacteria increased by 13%. Bacterial inactivation will be greater when the juice has more contact with plasma, as more contact results in more encounters with active species created by the plasma. Considering the results obtained and the fact that the nozzle gives an angle to the juice particles when spraying it, it should be expected that the greatest contact of the juice with the plasma occurs when the plasma jet is inclined at a 45° angle to the nozzle flow, rather than perpendicular to it. Therefore, finding the right balance is essential to achieve the best results.

Considering that the optical spectroscopy results show that the UV‐C emitted in this study is very low and that of the three ultraviolet regions, the UV‐C region has the ability to inactivate bacteria, with maximum inactivation occurring approximately in the range of 254 to 264 nm, the effect of ultraviolet radiation generated with the atmospheric pressure plasma jet designed in this study on inactivating *E. coli* present in cherry juice was considered negligible.

### 3.2 Effect of ACP‐PEF Treatment on the Qualitative Properties of Sour Cherry Juice

#### 3.2.1 Effect of ACP‐PEF Treatment on the TPC

Table 3 presents the ANOVA findings on the impact of ACP‐PEF treatment on TPC, TAC, and vitamin C levels. As shown in the table, all factors, except for two interactions (1: electric field exposure time*angle between plasma jet and nozzle (BD), 2: argon to air ratio*angle between plasma jet and nozzle (CD)), had a significant effect on TPC at the 10% probability level. The ANOVA's total sum of squares reveals that, among the primary variables, electric field intensity explains about 44% of the variance, making it the most influential factor in the reduction of TPC.

**TABLE 3:** Analysis of variance of the variables of electric field intensity, electric field exposure time, argon to air ratio and angle between plasma jet and nozzle on the qualitative properties (TPC, TAC, and vitamin C) in sour cherry juice.

Source	df	TPC (mg GAE/100 g)		TAC (mg C3/L)		Vitamin C (mg/L)	
		Sum of squares	Mean square	Sum of squares	Mean square	Sum of squares	Mean square
Model	14	3089.80	220.70^a^	1838.50	131.32^a^	333.61	23.83^a^
A	1	1346.20	1346.20^a^	607.66	607.66^a^	28.18	28.18^a^
B	1	347.76	347.76^a^	598.26	598.26^a^	10.58	10.58^a^
C	1	1027.86	1027.86^a^	375.45	375.45^a^	141.19	141.19^a^
D	1	174.04	174.04^a^	198.61	198.61^a^	148.41	148.41^a^
AB	1	4.00	4.00^a^	0.2500	0.2500^a^	1.56	1.56^a^
AC	1	0.2500	0.2500^a^	0.0007	0.0007^b^	0.0004	0.0004^b^
AD	1	1.0000	1.0000^a^	1.0000	1.0000^a^	0.8372	0.8372^a^
BC	1	0.2550	0.2550^a^	0.3660	0.3660^a^	0.0156	0.0156^b^
BD	1	0.0000	0.0000^b^	0.2603	0.2603^a^	1.72	1.72^a^
CD	1	0.0000	0.0000^b^	0.0100	0.0100^b^	0.3534	0.3534^a^
A^2^	1	157.91	157.91^a^	52.75	52.75^a^	0.0151	0.0151^b^
B^2^	1	54.57	54.57^a^	1.87	1.87^a^	0.3911	0.3911^a^
C^2^	1	2.68	2.68^a^	3.62	3.62^a^	0.0084	0.0084^b^
D^2^	1	4.90	4.90^a^	0.4587	0.4587^a^	0.4870	0.4870^a^
Residual	12	0.8318	0.0693	0.8792	0.0733	0.0979	0.0082
Lack of fit	10	0.5834	0.0583^b^	0.6818	0.0682^b^	0.0763	0.0076^b^
Pure error	2	0.2485	0.1242	0.1976	0.0988	0.0216	0.0108
Cor total	26	3090.63					

^a^ Significant effect at 10% level.

^b^ Not significant.

The model derived from the RSM for assessing the rate of change in TPC levels is a fully quadratic equation with a coefficient of determination (*R* ^2^) of 0.9987, a standard deviation (SD) of 0.26, and a coefficient of variation (C.V) of 0.1. The equation in coded form is presented in Equation (17), whereas Equation (18) represents the real model. In Equation (17), the electric field intensity factor has the largest negative coefficient, highlighting it as the most influential parameter in reducing TPC during the AC‐PEF treatment.
(17)

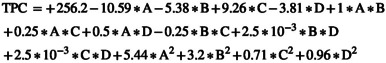



(18)

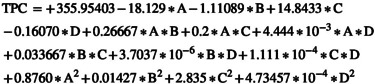




**FIGURE 4:**
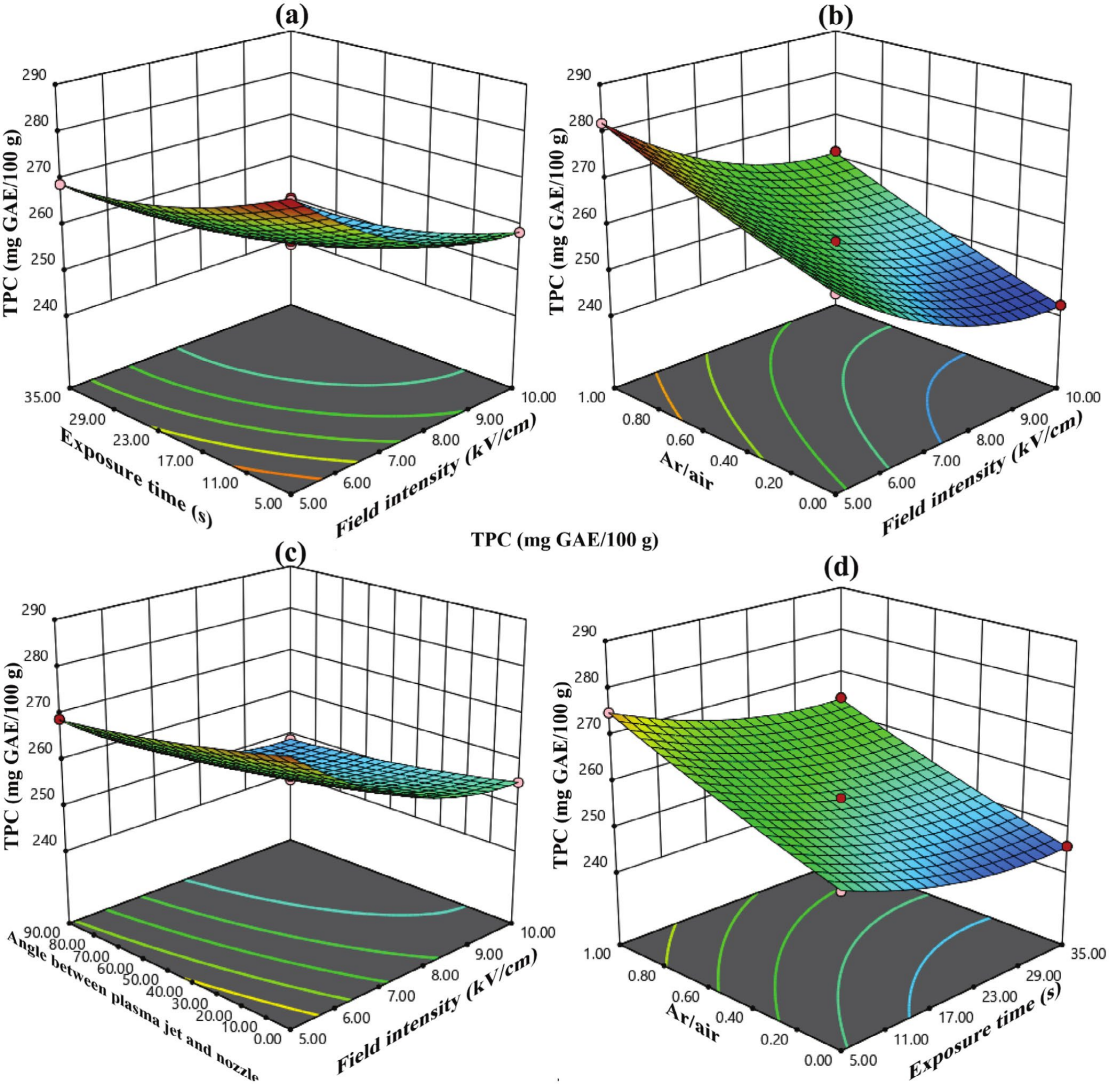
(a) The effect of electric field intensity and electric field exposure time, (b) the effect of electric field intensity and argon to air ratio, (c) the effect of electric field intensity and angle between plasma jet and nozzle, and (d) the effect of electric field exposure time and argon to air ratio on TPC level in sour cherry juice.

Figure 4 illustrates that as the electric field intensity, exposure time, and angle between the plasma jet and nozzle increase, the reduction in TPC becomes more pronounced during ACP‐PEF treatment. Among these factors, the electric field intensity has the most significant impact, with an 8% decrease in TPC when the intensity rises from 5 to 10 kV/cm. In contrast, when the exposure time increases from 5 to 35 s, the TPC reduction is only 4%. Additionally, increasing the angle between the plasma jet and the nozzle from 0° to 90° leads to a 3% decrease in TPC.

The reduction in TPC during ACP‐PEF treatment is primarily attributed to the oxidative effects of RONS generated by the plasma, which can degrade phenolic compounds through oxidation and polymerization reactions (Bigi et al. 2023). Additionally, PEF‐induced electroporation may enhance the exposure of intracellular phenolic compounds to these reactive species, accelerating their degradation. However, the observed 8% reduction in TPC at higher electric field intensities (10 kV/cm) is significantly lower than that caused by thermal pasteurization (16%), indicating that the ACP‐PEF system better preserves phenolic content (Hosseini et al. 2020). Variations in TPC response across studies, such as no significant change in sour cherry juice (Hosseini et al. 2020) or increases in pomegranate juice (Herceg et al. 2016), suggest that the impact depends on juice composition and treatment conditions.

#### 3.2.2 Effect of ACP‐PEF Treatment on the TAC

Table 3 reveals that all factors, except for two interactions (1: electric field intensity*argon to air ratio (AC), 2: argon to air ratio*angle between plasma jet and nozzle (CD)), had a significant impact on the TPC level at the 10% probability level. The ANOVA results indicate that, of the main variables, both electric field intensity and exposure time contributed roughly 33% each to the overall variation in the data.

The model derived from the RSM to assess the change rate in TAC levels is a complete quadratic model, exhibiting a coefficient of determination (*R* ^2^) of 0.9976, a standard deviation (SD) of 0.27, and a coefficient of variance (C.V.) of 0.12. The Equation for this model in coded form is presented in Equation (19), whereas the actual model is shown in Equation (20). In Equation (19), the factors with the largest negative coefficients are electric field intensity and exposure time, highlighting these variables as the primary contributors to the reduction of TAC through AC‐PEF treatment.
(19)

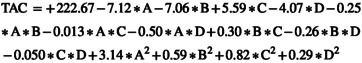



(20)

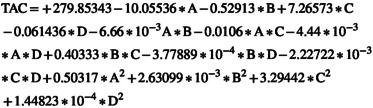




**FIGURE 5:**
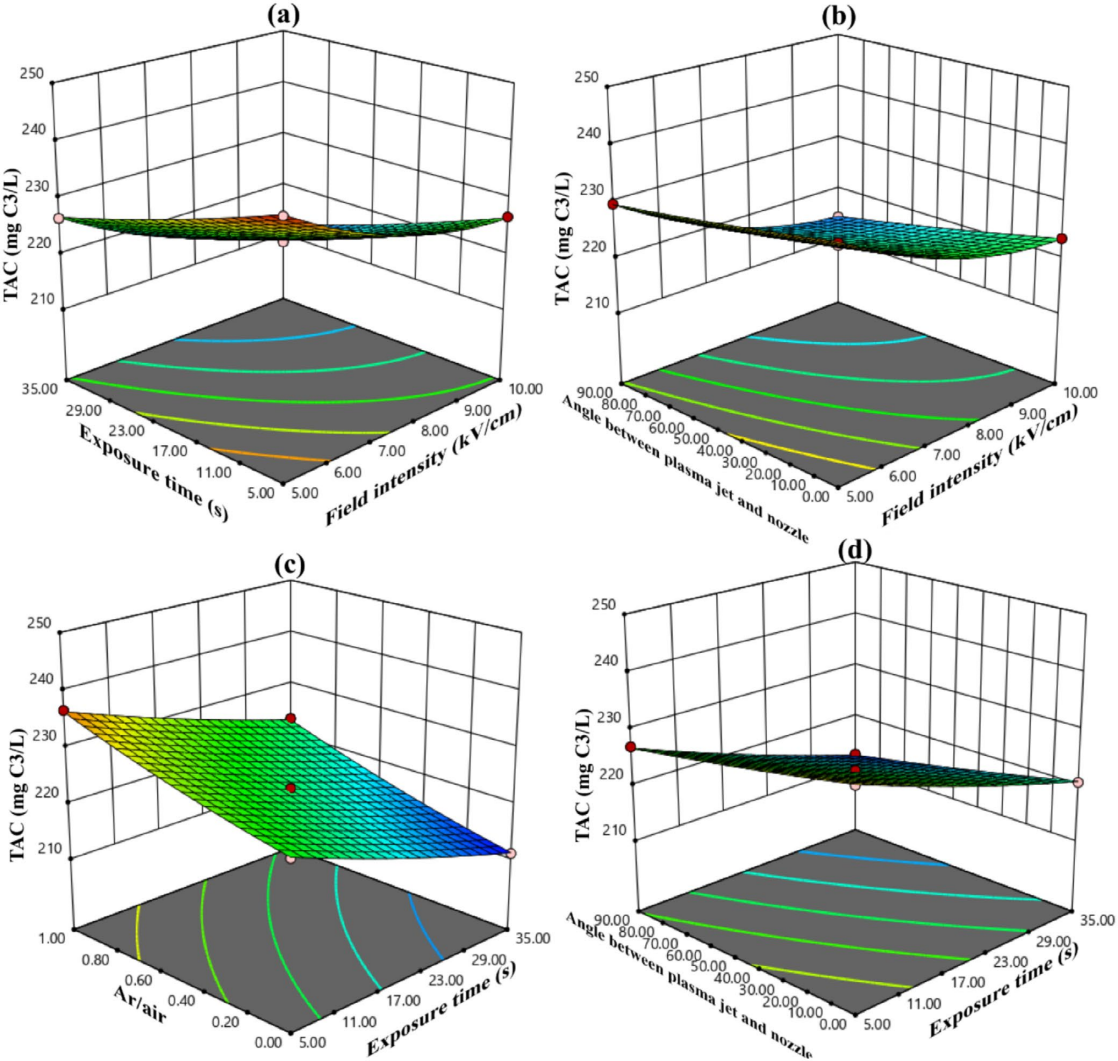
(a) The effect of electric field intensity and electric field exposure time, (b) the effect of electric field intensity and angle between plasma jet and nozzle, (c) the effect of electric field exposure time and argon to air ratio, and (d) the effect of electric field exposure time and angle between plasma jet and nozzle on TAC in sour cherry juice.

Figure 5 illustrates that as the electric field intensity, exposure time, and the angle between the plasma jet and nozzle increase, the reduction in TAC during ACP‐PEF treatment becomes more pronounced. The decrease in TAC during ACP‐PEF treatment is likely due to the oxidative degradation of anthocyanins by RONS, particularly hydroxyl radicals (OH) and ozone, which disrupt the conjugated structures of anthocyanins (Bigi et al. 2023). PEF‐induced electroporation may facilitate the interaction of these reactive species with anthocyanins, enhancing their breakdown. The 6% reduction in TAC at higher field intensities and exposure times (10 kV/cm, 35 s) is notably lower than the 16% reduction observed with thermal pasteurization, highlighting the advantage of the ACP‐PEF system in preserving anthocyanin content (Hosseini et al. 2020). The increase in TAC with higher argon‐to‐air ratios (5.51%) is attributed to reduced RONS concentrations, which minimizes oxidative stress on anthocyanins (Yepez et al. 2020).

#### 3.2.3 Effect of ACP‐PEF Treatment on the Vitamin C

Table 3 indicates that all factors, except for two interactions (1: electric field intensity*argon to air ratio (AC), 2: electric field exposure time*argon to air ratio (BC)), along with the squared terms for electric field intensity (A^2^) and argon to air ratio (C^2^), had a significant effect on vitamin C levels at the 10% probability level. The ANOVA results demonstrate that, of the main variables, the argon to air ratio and the angle between the plasma jet and nozzle together account for nearly 87% of the total variation in the data.

The RSM yielded a complete quadratic model for evaluating changes in vitamin C content, with a high determination coefficient (*R* ^2^) of 0.9985, a standard deviation (SD) of 0.09, and a coefficient of variation (C.V.) of 0.27. Equation (21) represents the model using coded variables, whereas Equation (22) displays the model in actual terms. Among the model coefficients in Equation (21), the most negative coefficient corresponds to the angle between the plasma jet and the nozzle, whereas the most positive coefficient is associated with the argon to air ratio. This indicates that these two parameters have the strongest influence on vitamin C variation during AC‐PEF treatment. Specifically, increasing the argon to air ratio helps preserve more vitamin C, whereas increasing the angle between the jet and the nozzle tends to result in greater vitamin C loss in cherry juice.
(21)

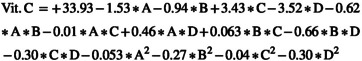



(22)

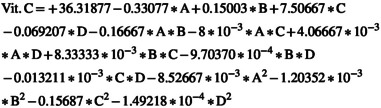




**FIGURE 6:**
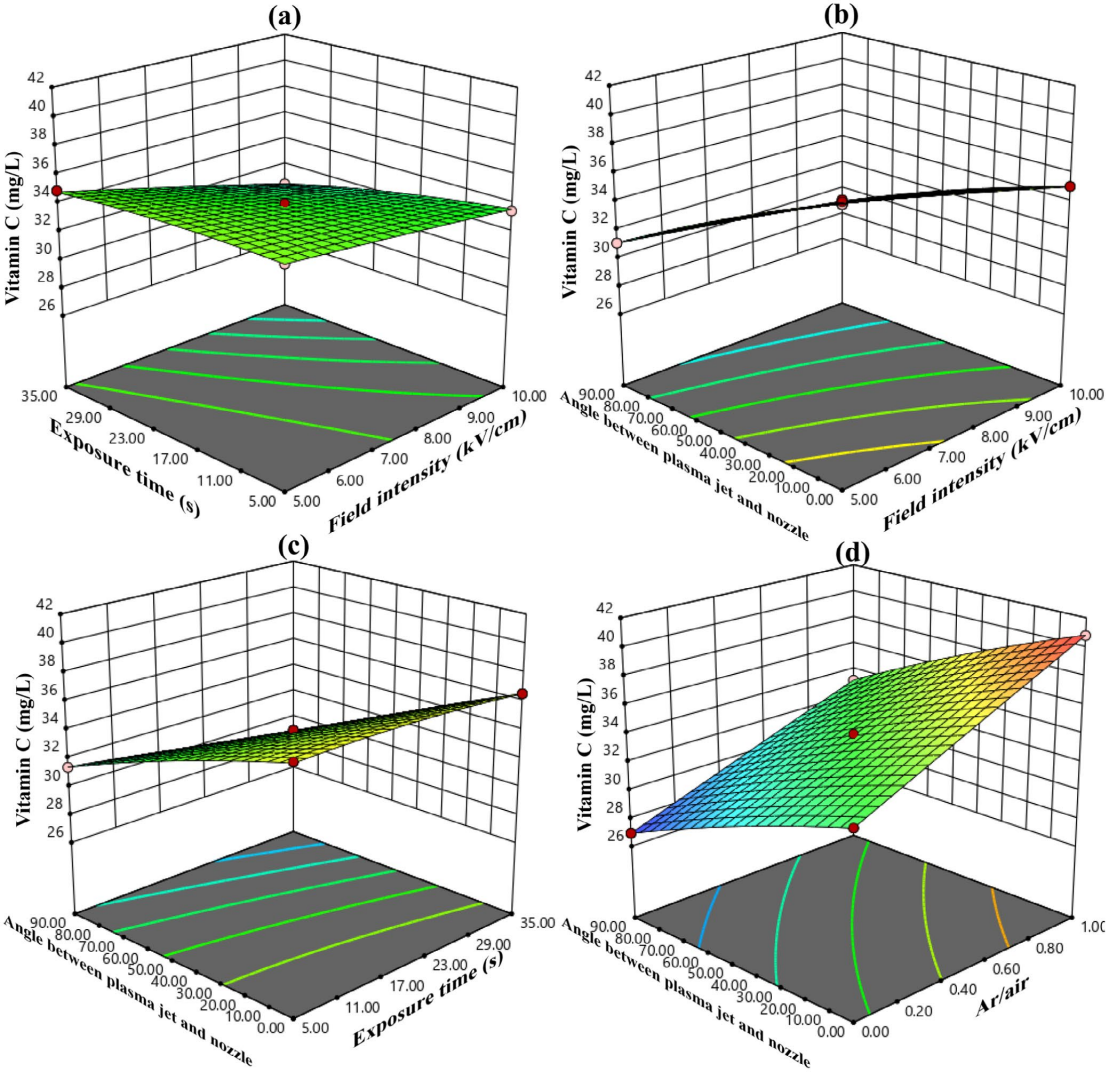
(a) The effect of electric field intensity and electric field exposure time, (b) the effect of electric field intensity and angle between plasma jet and nozzle, (c) the effect of electric field exposure time and angle between plasma jet and nozzle, and (d) the effect of electric field exposure time and argon to air ratio on vitamin C range in sour cherry juice.

Figure 6 demonstrates that as the electric field intensity, exposure time, and the angle between the plasma jet and nozzle increase, there is a greater reduction in vitamin C during ACP‐PEF treatment. Among these variables, the angle between the plasma jet and nozzle has the most pronounced impact. Specifically, when this angle shifts from 0° to 90°, vitamin C content drops by 19%. In comparison, an increase in field strength from 5 to 10 kV/cm results in an 8% decrease, whereas extending the exposure duration from 5 to 35 s leads to a 5% reduction.

Several studies have documented the loss of total anthocyanin content and vitamin C during both thermal and nonthermal processing methods. For instance, atmospheric pressure plasma has been applied for the pasteurization of strawberries (Misra et al. 2015), blueberry juice (Hou et al. 2019), and cherry juice (Hosseini et al. 2020). The breakdown of anthocyanins and ascorbic acid is likely attributed to oxidative mechanisms involving reactive species generated during plasma treatment. Ascorbic acid tends to degrade more readily than anthocyanins, which is mainly due to its inherent chemical and physical instability in the presence of reactive radicals (Hosseinzadeh Samani et al. 2016).

Figure 4b,d shows that by increasing the argon‐to‐air ratio, the TPC of sour cherry juice treated by the ACP‐PEF system also increased, such that the increasing argon‐to‐air ratio from 0 to 1 increased the TPC by 7.45%. According to Figure 5c, with increasing argon‐to‐air ratio, the TAC of sour cherry juice treated by the ACP‐PEF system also increased, such that with increasing argon‐to‐air ratio from 0 to 1, the TAC increased by 5.51%. Figure 6d also shows that as the argon‐to‐air ratio increases, the amount of vitamin C in the treated sour cherry juice treated by the ACP‐PEF system also increases, such that with increasing argon‐to‐air ratio from 0 to 1, the TAC increased by 23%. The reason for the increase in TPC, TAC, and vitamin C with increasing argon‐to‐air ratio is the decrease in ROS and RNS in the plasma produced with.

### 3.3 Optimization of ACP‐PEF System for Inactivation of *E. coli*

To achieve the highest reduction of *E. coli* in sour cherry juice, the ACP‐PEF system was optimized based on multiple parameters. The constraints used for this optimization process are outlined in Table 4. According to the results, the ideal settings within these limits for effective microbial inactivation were an electric field intensity of 10 kV/cm, an exposure time of 35 s, an argon‐to‐air ratio of 0.63, and an angle of 3.44° between the plasma jet and the nozzle. In this case, the ACP‐PEF system results in 5.73 log bacterial inactivation.

**TABLE 4:** Boundary conditions used for the optimization.

Name	Goal	Lower limit	Upper limit	Lower weight	Upper weight	Importance
Electric field intensity (kV/cm)	In range	5	10	1	1	3
Electric field exposure time (s)	In range	5	35	1	1	3
Argon/air	Minimize	0	1	1	1	3
Angle between plasma jet and nozzle	In range	0	90	1	1	3
Log (*N*/*N*_0_)	Minimize	−5.73	−1.55	1	10	5

Analyzing the outcomes of PEF and ACP treatments individually under their respective optimal conditions for eliminating *E. coli* in sour cherry juice, it becomes evident that integrating these two nonthermal methods through a hurdle technology approach by developing a combined ACP‐PEF system leads to a synergistic effect. This combined method resulted in greater bacterial inactivation than the sum of the effects observed when each treatment was applied separately under similar conditions. The enhanced effectiveness can be attributed to leveraging the strengths of both techniques while compensating for their limitations.

### 3.4 Comparison of PEF‐ACP Treatment Under Optimal Conditions With Conventional Thermal Pasteurization

Following ACP‐PEF treatment of sour cherry juice under optimized conditions, measurements were taken for TPC, TAC, vitamin C content, and color variation. These values were then analyzed in comparison to both the untreated sample and the sample subjected to conventional thermal processing. The comparative data are summarized in Table 5.

**TABLE 5:** Comparison of PEF‐ACP treatment under optimal conditions with conventional thermal pasteurization.

Samples	TPC (mg GAE/100 g)	TAC (mg C3/L)	Vitamin C (mg/L)	Color			
				b*	a*	L*	ΔE
Untreated	283.71	241.23	41.94	20.20	51.60	34.50	—
Thermal treated	237.11	201.57	19.62	19.30	49.80	31.20	3.86
ACP‐PEF treated	249.62	212.11	30.50	19.90	50.70	33.70	1.24

Based on the obtained results, it can be concluded that applying the ACP‐PEF system to sour cherry juice under optimal conditions not only effectively inactivates *E. coli* , but also results in fewer alterations to the juice's quality characteristics when compared to the conventional thermal treatment. Because ACP‐PEF treatment caused a 12% reduction in TPC, a 12% reduction in TAC, and a 27% reduction in vitamin C compared to the untreated sample, whereas thermal treatment caused a 16% reduction in TPC, a 16% reduction in TAC, and a 53% reduction in vitamin C compared to the untreated sample. Also, the color changes resulting from Equation (20) in sour cherry juice treated ACP‐PEF system are much less than the color changes in sour cherry juice treated with a conventional thermal method because ACP‐PEF treatment caused a 2.3% reduction in lightness value, a 1.7% reduction in redness value, and a 1.5% reduction in yellowness value compared to the untreated sample, whereas thermal treatment caused a 9.6% reduction in lightness value, a 3.5% reduction in redness value, and a 4.5% reduction in yellowness value compared to the untreated sample.

Other studies have also reported that nonthermal methods, whether used alone or in combination, tend to cause less alteration in the quality and appearance of food products compared to conventional thermal methods. For instance, one study found that plasma treatment had no significant impact on the color of blueberry juice at the 5% significance level, with the color of treated samples remaining very similar to that of the untreated ones, unlike thermal‐treated samples (Hou et al. 2019). Additionally, a separate investigation evaluated the effects of two combined technologies, ultrasound‐assisted PEF and hydrodynamic pulsed electric field, on pasteurizing sour cherry juice. The findings revealed that these hybrid nonthermal approaches maintained the juice's quality attributes more effectively than traditional heat‐based processing (Hosseini et al. 2020).

## 4 Conclusion

The decontamination of sour cherry juice using the ACP‐PEF system demonstrated that the electric field exposure time is the most significant factor influencing the inactivation of *E. coli* . A longer exposure time enhances the inactivation through the PEF itself and creates more favorable conditions for plasma free radicals to interact with bacterial components due to increased electroporation. When comparing the effects of the PEF, ACP, and ACP‐PEF systems on *E. coli* inactivation, it was observed that the combination of PEF and ACP technologies produced a synergistic effect. Additionally, a comparison of ACP‐PEF treatment under optimal conditions with conventional thermal treatment revealed that the combined system not only inactivated bacteria more effectively but also better preserved the quality and color of sour cherry juice. As a result, ACP‐PEF emerges as a promising alternative to traditional thermal pasteurization methods.

## Author Contributions

**Farhad Jamali:** investigation (equal), methodology (equal), writing – original draft (equal), writing – review and editing (equal). **Bahram Hosseinzadeh Samani:** conceptualization (equal), project administration (equal), supervision (equal), writing – review and editing (equal). **Kimia Taki:** writing – review and editing (equal). **Shirin Ghatrehsamani:** writing – review and editing (equal).

## Conflicts of Interest

The authors declare no conflicts of interest.

## Data Availability

Data is available on request from the authors.
